# Complete chloroplast genome of the marine eelgrass *Zostera pacifica* (Zosteraceae, Plantae) from Monterey, California

**DOI:** 10.1128/mra.01189-24

**Published:** 2025-01-15

**Authors:** Sosan Abdulrahman, Adilene Aguirre, Eliana Arriaga, Ashley C. Avina, Angeles Badajos, Yureni A. Badillo, Alexis M. Bañuelos, Javier Pulido, Bet-sua Bucio Valdovinos, Taliyah Champaco, Rodrigo Chavez, Gregorio Chavez-Felix, Melaina Cruz, Melissa Distancia, Kingsley Dzubay, Andres Espinoza, Julie Estrada, Danneli Fernandez, Alejandra Gutierrez-Villanueva, Marc Guzman, Jimena Hernandez-Tejeda, Victor Hernandez, Jeffery R. Hughey, Sebastian Jalomo, Hanna Khanaka, Juliah Kuepfer, Alyssa M. Laurel, Julia Lewis, Hanah E. Lopez, Ana Martinez, Michell J. Martinez, Jorge A. Medina Ramirez, Cesar M. Ramirez, Fatima Mendez, Ashley Mendoza-Torralba, Kajal Naidu, Abraham Nolasco, Lupita Nuñez, Vivian Paredes, Jesse P. Anaya, Sreenivas Rajesh, Kevin Ramirez, Andrea Salazar, Jeriel S. Sevilla, Jennifer G. Tijero, Aaron Tinoco Viorato, Heaven Valdez, on behalf of Hartnell College Genomics Group

**Affiliations:** 1 Division of Mathematics, Science, and Engineering, Hartnell College, Salinas, California, USA; University of Strathclyde, Glasgow, United Kingdom

**Keywords:** bioinformatics, high-throughput sequencing, plastid genome, seagrass, *Zostera marina*

## Abstract

We present the complete chloroplast genome of the eelgrass *Zostera pacifica* from Monterey, California. The genome is circular and 144,675  bp in length. It consists of 82 protein-coding, 31 transfer RNA, and 8 ribosomal RNA genes and is 99.44%–99.42% similar in nucleotide pairwise identity to the closely related species *Zostera marina*.

## ANNOUNCEMENT

*Zostera* L. is a genus of seagrass with worldwide distribution and great ecological importance (1). Five species are currently recognized in the genus (2), including *Zostera pacifica* S.Watson, a wide-leafed subtidal species originally described from Puget Sound, Washington, Santa Barbara, and Monterey, California (3). Genetic marker analyses show that *Z. pacifica* is distinct from, but very closely related to *Zostera marina* L. (4, 5). Although there have been numerous investigations of the nuclear and organellar genomes of *Z. marina* (6–9), no genomes of *Z. pacifica* have been published. To contribute to the systematics of *Zostera*, the complete chloroplast genome of topotype *Z. pacifica* was assembled and characterized.

The specimen of *Z. pacifica* was collected from Del Monte Beach, Monterey, California (decimal degrees 36.601175, −121.885193), voucher number Hartnell College Collection #273 (Salinas, California). The DNA was extracted from leaf material using the DNeasy Blood and Tissue Kit (Qiagen) following the manufacturer’s protocol with two modifications: the binding step was 4,000 × *g* for 3 minutes, and the DNA was eluted in 40 µL Tris-Acetate-EDTA (TAE) after 7 minutes of incubation (10). The 150 bp paired-end library was constructed with the NEBNext Ultra II DNA Library Prep Kit (New England BioLabs) and sequenced on an Illumina NovaSeq 6000 (Illumina, Inc.) by Novogene Corporation Inc. The sequencing generated 27,854,284 reads that were filtered using default BBDuk 1.0 (11) settings in Geneious Prime 2019.1.3 (Biomatters Limited). The chloroplast genome was assembled with GetOrganelle 1.7.5+ using the trimmed reads and default settings (12). The assembly recovered a single chloroplast contig with an average coverage of 4,059×. The draft genome was confirmed for accuracy with the map to reference function in Geneious Prime using the Low Sensitivity/Fastest setting and three iterations. The annotation was performed with the online annotator CPGAVAS2 (http://47.96.249.172:16019/analyzer/home) (13) designating *Nanozostera japonica* (Ascherson & Graebner) Tomlinson & Posluszny (GenBank accession number NC_058623) (14) as the reference using the default settings and manually adjusting start and stop positions according to NCBI ORFfinder (https://www.ncbi.nlm.nih.gov/orffinder/) and Sequin 15.5 (15). Nucleotide identities were calculated by BLAST+ 2.15.0 searches using the default settings (16).

**Fig 1:**
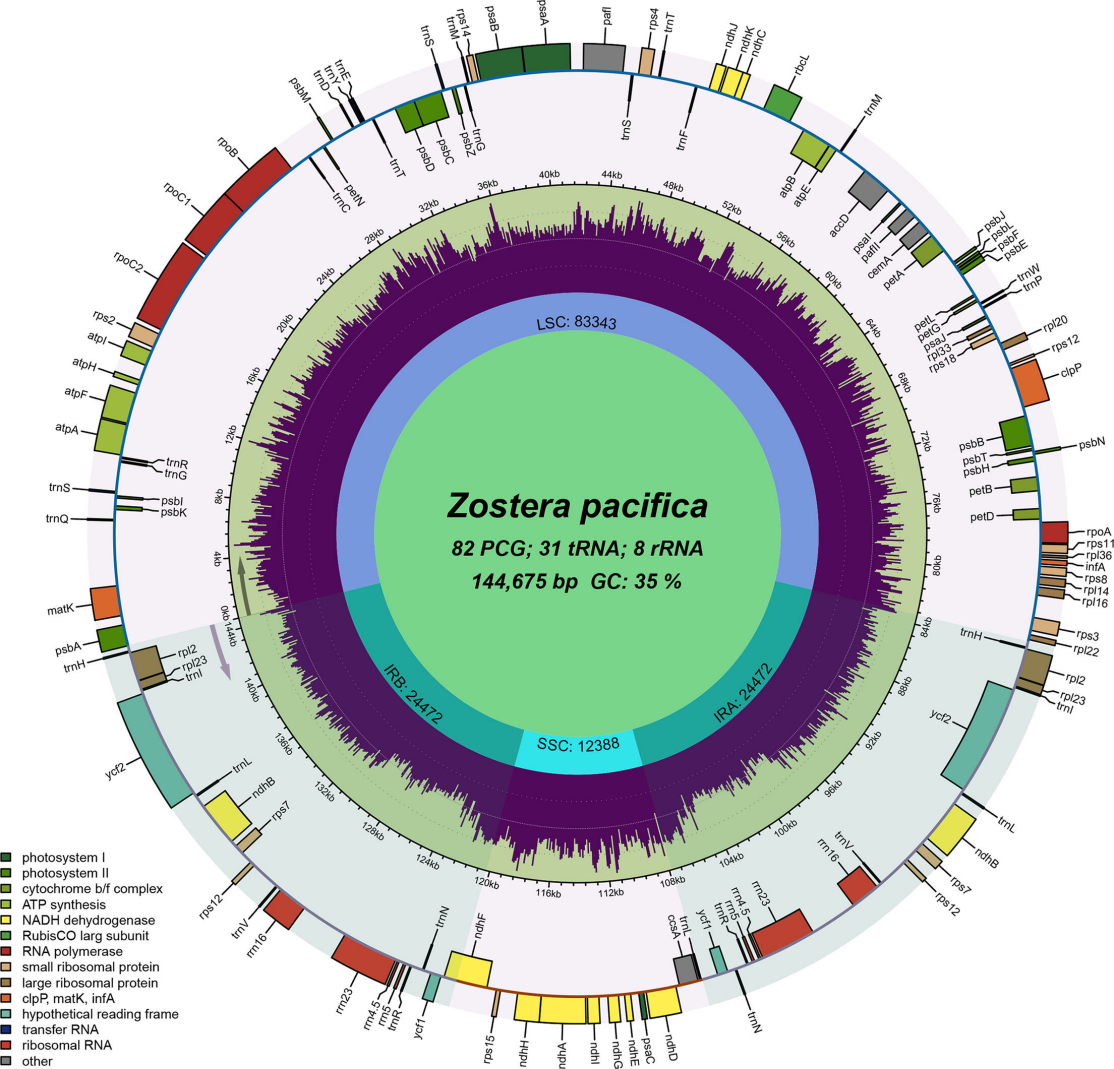
Complete chloroplast genome map of *Zostera pacifica*. The genome was annotated using CPGAVAS2 (13) and mapped with Chloroplot 0.2.4 (18). The innermost ring displays the AT content in purple color and the direction of transcription, as indicated by the arrows. The final ring displays the genes. Genes transcribed clockwise are on the inside, while counterclockwise transcriptions are positioned on the outside. The color coding corresponds to genes of different groups as listed in the key in the bottom left.

The complete circular chloroplast genome of *Z. pacifica* is 144,675 bp in length and has an Adenine Thymine (AT%) skew of 65.0% (Fig. 1). The genome displays the characteristic higher plant organization which includes two identical inverted repeats (IRs) separated by one large single copy (LSC) section and one small single copy (SSC) section (17). The IRs, LSC, and SSC are 24,472, 83,343, and 12,388 bp in length, respectively. The genome contains 121 genes including 82 protein-coding, 31 tRNA, and 8 rRNA genes (Fig. 1). Genome length, content, and structure are comparable to previously published Zosteraceae (Table 1) (6, 9, 14). The chloroplast genome sequence of *Z. pacifica* is 99.44% and 99.42% similar to the genomes of *Z. marina* from Finland (not accessioned) (6) and China (GenBank accession number NC_036014) (9), respectively. These nucleotide identities are equivalent to the congeners *Nanozostera muelleri* (Irmisch ex Ascherson) Tomlinson & Posluszny and *N. japonica*, which are 99.34% similar in sequence (14).

**TABLE 1:** Chloroplast genome characteristics of *Zostera pacifica* and other published Zosteraceae^*a*^

Species	Genome size (bp)	LSC size (bp)	IR size (bp)	SSC size (bp)	GC, %^*b*^	PCG	tRNA	rRNA	Total genes	GenBank	Reference
*Heterozostera nigricaulis*	145,883	83,541	24,430	13,482	35.9	85	38	8	131	NC_058621.1	(14)
*Nanozostera japonica*	146,090	83,664	24,628	13,169	35.9	85	34	8	127	NC_058623.1	(14)
*Nanozostera muelleri*	145,820	83,524	24,399	13,498	35.9	85	38	8	131	NC_068805.1	(14)
*Phyllospadix iwatensis*	152,726	84,753	25,167	17,639	36.2	86	38	8	132	NC_058622.1	(14)
*Zostera marina*, China	143,877	83,224	25,915	8,823	35.5	78	30	8	116	NC_036014.1	(9)
*Zostera marina*, Finland	143,968	83,312	24,127	12,402	35	82	34	8	124	None	(6)
*Zostera pacifica*	144,675	83,343	24,472	12,388	35	82	31	8	121	PQ286553.1	This study

^*a*^ PCG, protein coding genes; bp, base pairs; tRNA, transfer RNA genes; rRNA, ribosomal RNA genes.

^*b*^ GC, Guanine Cytosine.

## Data Availability

The complete chloroplast genome sequence of *Zostera pacifica* is available in GenBank under accession number PQ286553. The associated BioProject, SRA, and BioSample numbers are PRJNA1176131, SRS22972521, and SAMN44381036, respectively. The chloroplast genomes referenced in the text are *Nanozostera japonica* (GenBank accession number NC_058623) and *Z. marina* (GenBank accession number NC_036014).

## References

[1] Moore KA, Short FT. 2006. Seagrasses: biology, ecology and conservation, p 361–386. In Zostera: biology, ecology and management. Springer, Netherlands.

[2] Sullivan BK, Short FT. 2023. Taxonomic revisions in Zosteraceae (Zostera, Nanozostera, Heterozostera and Phyllospadix). Aquat Bot 187:103636. doi:10.1016/j.aquabot.2023.103636

[3] Watson S. 1890. Contributions to American botany. Proc Am Acad Arts Sci 26:124. doi:10.2307/20013481

[4] Coyer JA, Miller KA, Engle JM, Veldsink J, Cabello-Pasini A, Stam WT, Olsen JL. 2008. Eelgrass meadows in the California Channel Islands and adjacent coast reveal a mosaic of two species, evidence for introgression and variable clonality. Ann Bot 101:73–87. doi:10.1093/aob/mcm288 18006507 PMC2701847

[5] Coyer JA, Hoarau G, Kuo J, Tronholm A, Veldsink J, Olsen JL. 2013. Phylogeny and temporal divergence of the seagrass family Zosteraceae using one nuclear and three chloroplast loci. Syst Biodivers 11:271–284. doi:10.1080/14772000.2013.821187

[6] Olsen JL, Rouzé P, Verhelst B, Lin Y-C, Bayer T, Collen J, Dattolo E, De Paoli E, Dittami S, Maumus F, et al.. 2016. The genome of the seagrass Zostera marina reveals angiosperm adaptation to the sea. Nature New Biol 530:331–335. doi:10.1038/nature16548 26814964

[7] Ma X, Olsen JL, Reusch TBH, Procaccini G, Kudrna D, Williams M, Grimwood J, Rajasekar S, Jenkins J, Schmutz J, Van de Peer Y. 2021. Improved chromosome-level genome assembly and annotation of the seagrass, Zostera marina (eelgrass). F1000Res 10:289. doi:10.12688/f1000research.38156.1 34621505 PMC8482049

[8] Yu L, Khachaturyan M, Matschiner M, Healey A, Bauer D, Cameron B, Cusson M, Emmett Duffy J, Joel Fodrie F, Gill D, et al.. 2023. Ocean current patterns drive the worldwide colonization of eelgrass (Zostera marina). Nat Plants 9:1207–1220. doi:10.1038/s41477-023-01464-3 37474781 PMC10435387

[9] Xing Q, Guo J. 2018. Characterization of the complete chloroplast genome of the seagrass Zostera marina using Illumina sequencing technology. Conserv Genet Resour 10:419–422. doi:10.1007/s12686-017-0839-5

[10] Garcia AN, Ramos JH, Mendoza AG, Muhrram A, Vidauri JM, Hughey JR, Hartnell College Genomics Group. 2022. Complete chloroplast genome of topotype material of the coast live Oak Quercus agrifolia Née var. agrifolia (Fagaceae) from California. Microbiol Resour Announc 11:e0000422. doi:10.1128/mra.00004-22 35254126 PMC9022551

[11] Bushnell B. 2014. BBMap: a fast, accurate, splice-aware aligner. United States. Available from: https://www.osti.gov/biblio/1241166. Retrieved 16 Apr 2024.

[12] Jin J-J, Yu W-B, Yang J-B, Song Y, dePamphilis CW, Yi T-S, Li D-Z. 2020. GetOrganelle: a fast and versatile toolkit for accurate de novo assembly of organelle genomes. Genome Biol 21:241. doi:10.1186/s13059-020-02154-5 32912315 PMC7488116

[13] Shi L, Chen H, Jiang M, Wang L, Wu X, Huang L, Liu C. 2019. CPGAVAS2, an integrated plastome sequence annotator and analyzer. Nucleic Acids Res 47:W65–W73. doi:10.1093/nar/gkz345 31066451 PMC6602467

[14] Chen J, Zang Y, Shang S, Yang Z, Liang S, Xue S, Wang Y, Tang X. 2023. Chloroplast genomic comparison provides insights into the evolution of seagrasses. BMC Plant Biol 23:104. doi:10.1186/s12870-023-04119-9 36814193 PMC9945681

[15] Benson DA, Cavanaugh M, Clark K, Karsch-Mizrachi I, Ostell J, Pruitt KD, Sayers EW. 2018. GenBank. Nucleic Acids Res 46:D41–D47. doi:10.1093/nar/gkx1094 29140468 PMC5753231

[16] Camacho C, Coulouris G, Avagyan V, Ma N, Papadopoulos J, Bealer K, Madden TL. 2009. BLAST+: architecture and applications. BMC Bioinformatics 10:421. doi:10.1186/1471-2105-10-421 20003500 PMC2803857

[17] Dobrogojski J, Adamiec M, Luciński R. 2020. The chloroplast genome: a review. Acta Physiol Plant 42:98. doi:10.1007/s11738-020-03089-x

[18] Zheng S, Poczai P, Hyvönen J, Tang J, Amiryousefi A. 2020. Chloroplot: an online program for the versatile plotting of organelle genomes. Front Genet 11:576124. doi:10.3389/fgene.2020.576124 33101394 PMC7545089

